# Supplementary material for: Interfacial Charge Transfer Enhances Transient Surface Photovoltage in Hybrid Heterojunctions
Source: Nanomaterials (Basel). 2025 Jan 21;15(3):154. doi: 10.3390/nano15030154 (PMC11819976; doi:10.3390/nano15030154)
Supplement: Supplementary file 1 [file nanomaterials-15-00154-s001.zip › nanomaterials-3367372-supplementary.pdf]

SUPPORTING INFORMATION

Interfacial Charge Transfer Enhances Transient Surface Photovoltage in Hybrid Heterojunctions

Cristian Soncini[1,2], *Roberto Costantini*[1,3], *Martina Dell'Angela*[1], *Alberto Morgante*[1,3] *and Maddalena Pedio*[1,4].

[1] CNR - Istituto Officina dei Materiali (IOM), S.S. 14 km 163.5, 34149, Trieste, Italy.

[2] Elettra-Sincrotrone Trieste S.C.p.A., S.S. 14 km 163.5, 34149, Trieste, Italy.

[3] Dipartimento di Fisica, Università di Trieste, Via Valerio 2, 34127 Trieste, Italy.

[4] CNR - Istituto Officina dei Materiali (IOM), Via Pascoli, 06123, Perugia, Italy.

S1. PES spectra analysis

The Si 2p and C 1s core levels were monitored after each step of CuPc deposition. The spectra were fitted using Gaussian-Lorentzian components with a Lorentzian width of 0.11 eV and 0.14 eV for the Si 2p and C 1s core levels, respectively. The two main components of the Si 2p core level (Fig. S1), located at 99.2 eV and 103.4 eV of binding energy (BE), correspond to the crystalline Si and SiO_2 oxide layer, respectively. The crystalline Si component presents the typical spin-orbit splitting of the 2p core level ($2p_{3/2}$ and $2p_{1/2}$). The additional components in the fit correspond to SiO_x sub-oxide species (Si^+, Si^{2+}, Si^{3+}). Table 1 summarizes the best-fit parameters of the Si 2p spectra.

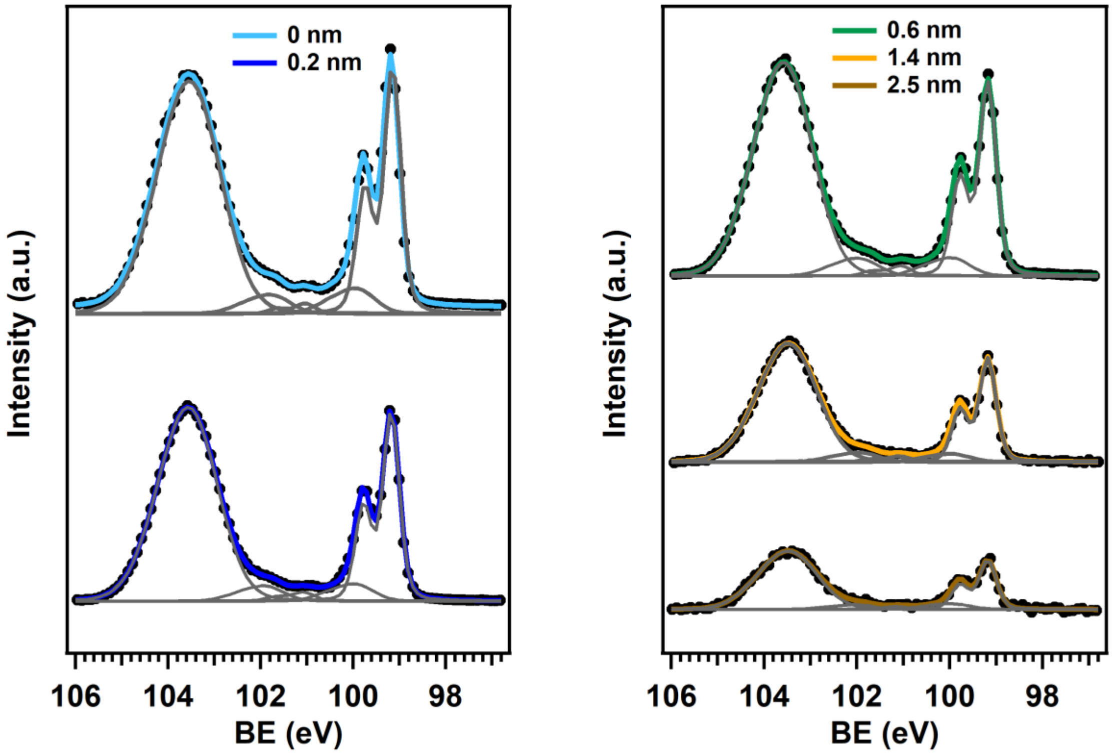

Figure S1. *Si 2p PES spectra as a function of the CuPc film thickness*

Peak	BE (eV)	A	GW (eV)	σ (nm)
Si $2p_{3/2}$	99.18	3.67	0.36	0
Si_{Ox}	103.39	8.22	1.29	
Si $2p_{3/2}$	99.18	2.79	0.36	0.2
Si_{Ox}	103.40	6.45	1.27	
Si $2p_{3/2}$	99.18	1.81	0.36	0.6
Si_{Ox}	103.42	4.34	1.25	
Si $2p_{3/2}$	99.18	0.79	0.36	1.4
Si_{Ox}	103.32	2	1.23	
Si $2p_{3/2}$	99.18	0.28	0.35	2.5
Si_{Ox}	103.33	0.73	1.2	

Table S1. *Best-fit parameters of the Si 2p spectra as a function of the CuPc film thickness. BE is the binding energy, A is the fit area (a.u.), GW is the Gaussian width and σ is the CuPc nominal thickness. In the fits has been used a Lorentzian width of 0.11 eV. The $2p_{1/2}$ BE (not reported) is located at 0.6 eV toward higher BE to the $2p_{3/2}$ core level in the table.*

The C 1s line shape is accurately reproduced using five components in the fit. The C_α and C_β components belong respectively to the inequivalent benzene and pyrrolic carbon rings, while the S_α and S_β components are the relative shake-up satellites.[1,2,3] The C_H component is

attributed to the in-plane excitation of the C-H stretching mode.[2,3] Table 1 summarizes the best-fit parameters of the C 1s core levels for increasing CuPc coverage.

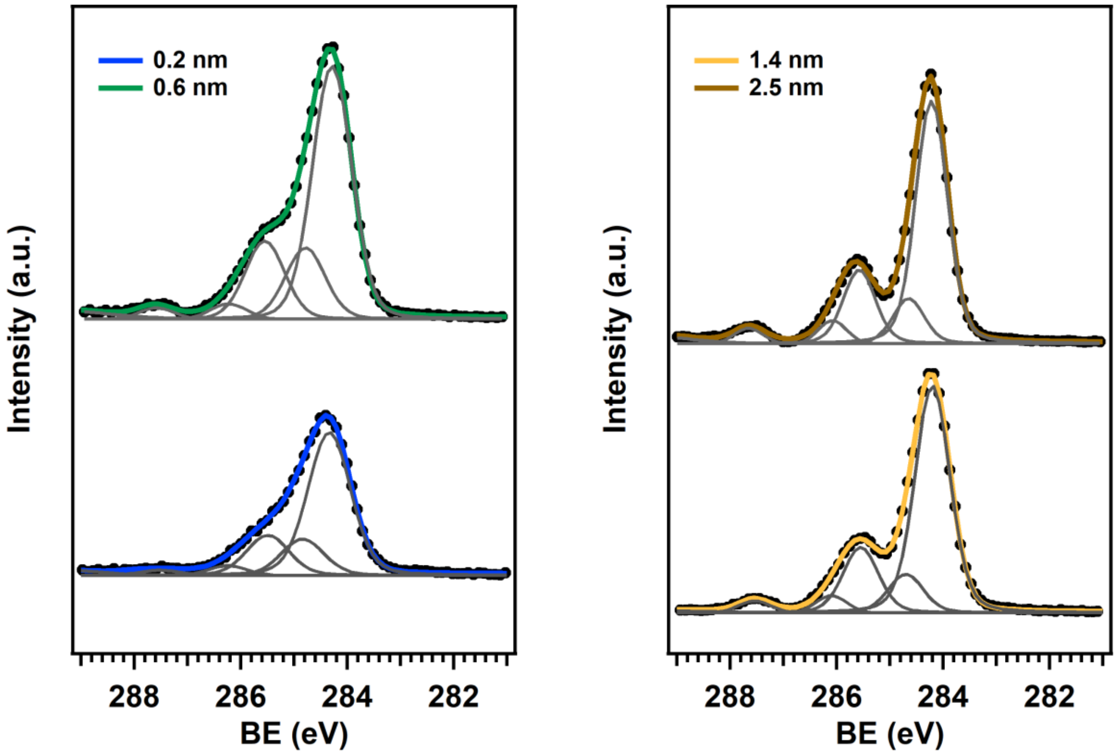

Figure S2. *C 1s PES spectra as a function of the CuPc film thickness.*

Peak	BE (eV)	A	GW (eV)	σ (nm)
C_α	284.34	1	0.82	0.2 nm
C_H	284.84	0.26	0.82	
C_β	285.45	0.29	0.86	
S_α	286.25	0.06*	0.85	
S_β	287.53	0.12*	0.88	
C_α	284.29	1	0.80	0.6 nm
C_H	284.79	0.29	0.80	
C_β	285.55	0.30	0.79	
S_α	286.23	0.05*	0.82	
S_β	287.59	0.13*	0.77	
C_α	284.20	1	0.67	1.4 nm
C_H	284.70	0.17	0.67	
C_β	285.55	0.29	0.67	
S_α	286.10	0.06*	0.68	
S_β	287.54	0.15*	0.72	
C_α	284.22	1	0.61	2.5 nm
C_H	284.65	0.18	0.60	
C_β	285.58	0.31	0.61	
S_α	286.08	0.07*	0.61	
S_β	287.66	0.17*	0.63	

Table S2. *Best-fit parameters of the C 1s spectra as a function of the CuPc film thickness. BE is the binding energy, A is the fit area normalized to C_{α}, GW is the Gaussian width and σ is the CuPc nominal thickness. In the fits has been used a Lorentzian width of 0.15 eV. *The shake-up satellite (S_x) area has been normalized to the relative C component (C_x) area.*

The nominal thicknesses of the oxide layer and CuPc films have been calculated according to the equation:[4]

$$\frac{I_A}{I_B} = K \frac{1 - \exp\left(-\frac{d}{\lambda_A \cos\theta}\right)}{\exp\left(-\frac{d}{\lambda_B \cos\theta}\right)} \qquad \text{(eq. S1)}$$

With:

$$K = \frac{I_A^{\infty}}{I_B^{\infty}}$$

Where d is the overlayer thickness, λ is the inelastic mean free path, θ is the electron take-off angle, K is the area ratio between an thick overlayer (> 3λ) and the clean substrate, and I_A and I_B are the total area of the core levels of the overlayer and substrate, respectively. To calculate the nominal thickness of the oxide layer we referred to K values from ref. 5.

From the Si 2p core level of the clean substrate, we completely reconstructed the energy level alignment of the SiO_2/p-Si(100) surface (Fig. 1c main text). The band bending (V_B) was determined following the equation:

$$V_B = (E_F - E_V) - (E_F - E_{VBM}) \qquad \text{(eq. S2)}$$

with:

$$(E_F - E_{VBM}) = E_{Si2p_{3/2}} - 98.74 \text{ eV}$$

Where 98.74 eV is the energy difference between the valence band maximum (E_{VBM}) and the $Si2p_{3/2}$ core level.[6] Since XPS is a surface-sensitive technique, $E_F - E_V$ (where E_V is the energy position of the valence band in the bulk) was calculated according to:[7]

$$(E_F - E_V) = K_B T \ln\left(\frac{N_V}{N}\right) \qquad \text{(eq. S3)}$$

Where N_v is the effective density of states in the valence band and N is the density of acceptors.

The continuous decrease of the C 1s peak broadening and increase of the C_α-C_β energy separation is in agreement with the growth found in ref. 8 on etched Si substrates, suggesting the presence of molecule-substrate interaction effects during the formation of the interface (Fig. S3). As the film thickness increases (bulk phase formation) molecules reorient toward the standing configuration.

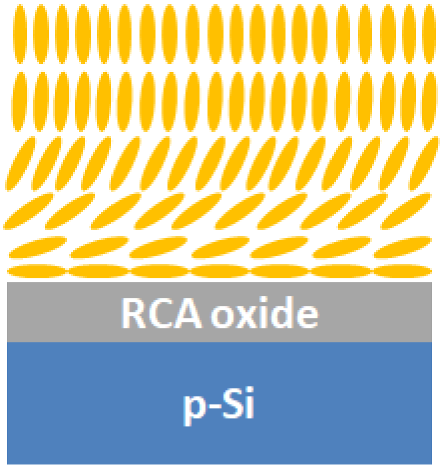

Figure S3. *Proposed scheme of CuPc growth on well-defined SiO_2/p-Si substrate.* [8]*It is worth noting that in case of CuPc on native SiO_2/Si the molecules in the first layers present a disordered adsorption.*

S2. Heterojunction's TR-PES spectra analysis

The intensity and photo-saturation condition of the SPV were monitored as a function of the pump fluence. Fig. S3 shows the energy shift of the Si $2p_{3/2}$ core level after excitation using different pump fluences. The SPV-induced energy shift saturates using a pump fluence of 0.46 μJ cm^{-2} for both the clean substrate and the heterojuntion. The apparent decrease in the Si 2p intensity is related to peak broadening induced by laser thermal effects. As estimated by the fitting procedure, the Si 2p peak areas before and after excitation are identical.

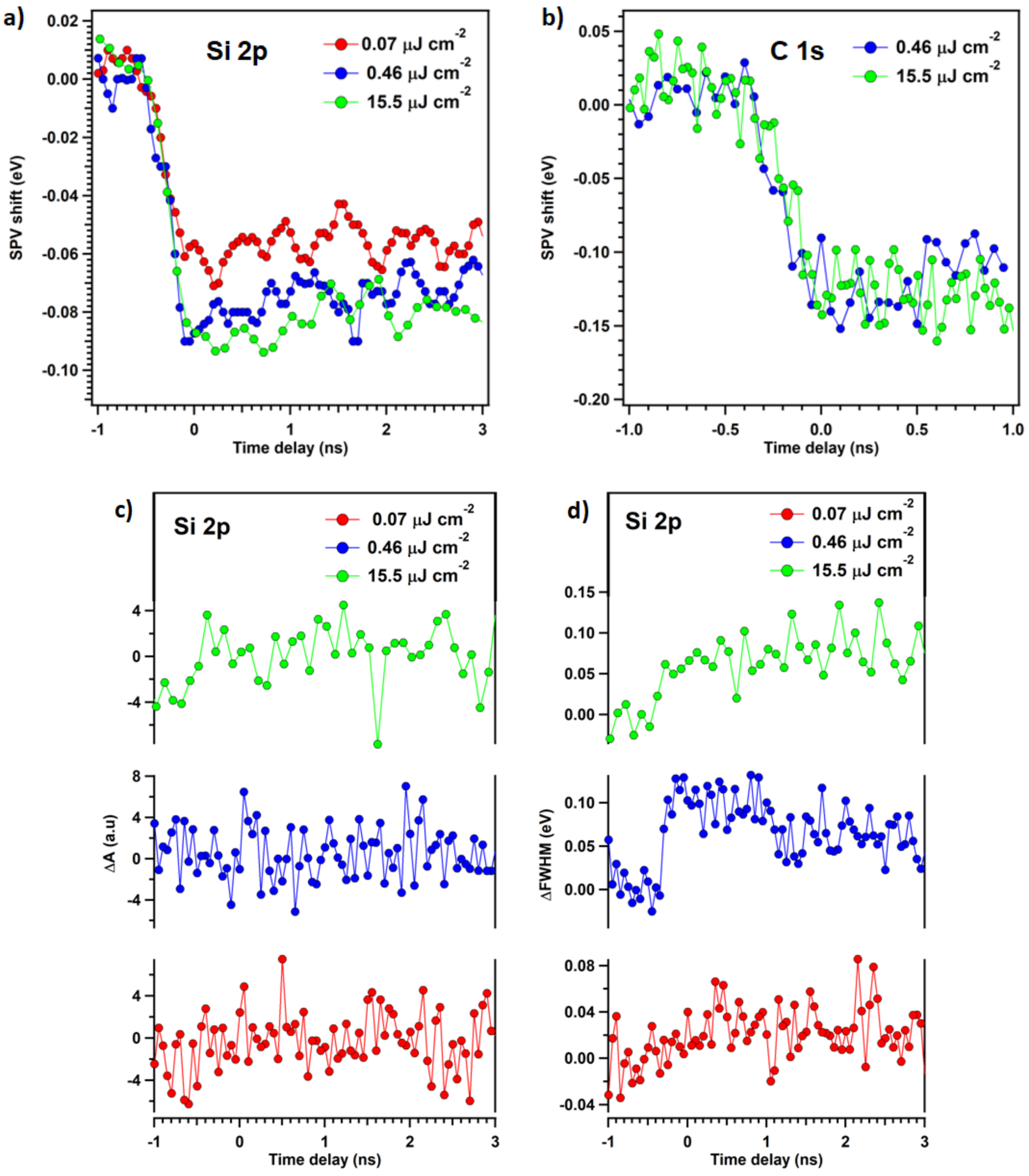

Figure S4. *SPV-induced energy shift of the a) Si 2p (clean substrate) and b) C 1s core levels as a function of the pump fluence; Differential (pump ON - pump OFF) Si 2p core level a) area and d) FWHM of the clean substrate, estimated by fitting the TR-PES data.*

Since the SPV dynamics do not fully relax within the experimental time window (Figure 2c main text), we checked for pile-up effects, i.e. the observed SPV signal may be lower than the actual one. Fig. S4 shows two measurements performed in TR-PES mode integrating the electron bunches of the synchrotron without laser excitation and between two consecutive laser pulses (period of 2.6 μs). The Si 2p core level shows a residual energy shift of 20 meV due to the incomplete recovering of the SPV dynamics between excitation pulses.

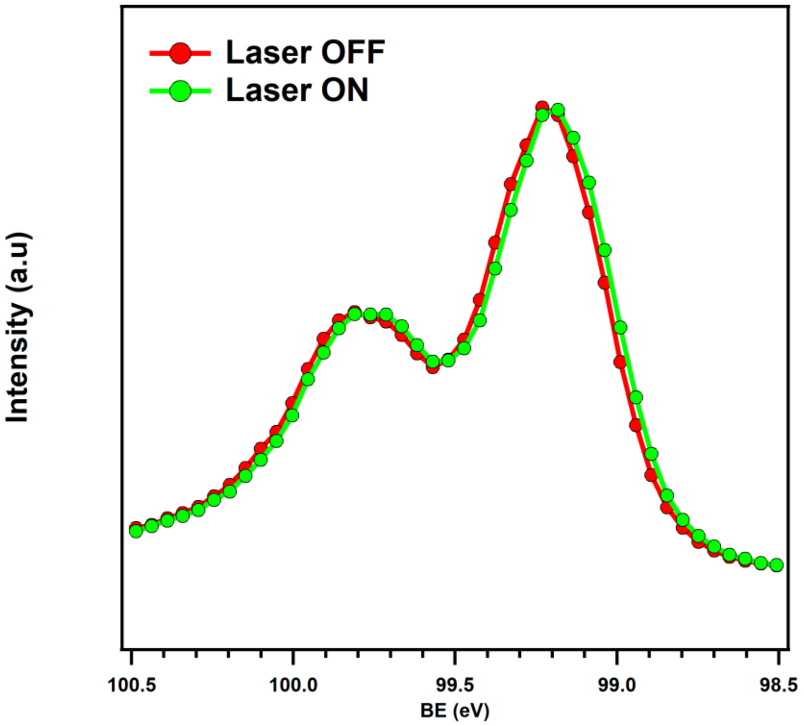

Figure S5. *Si 2p spectra performed in TR-PES mode and integrating the electron bunches of the synchrotron with and without laser excitation.*

S3 SPV Modelling

The SPV relaxation curves have been modelled following the thermionic model. For a detailed description of the model and mathematical derivation of equations refer to ref. 9. It considers only the SPV induced by excitation of surface/interface states and photon absorbed in the space charge region, assuming negligible the contribution from the bulk (diffusion of photo-generated free electrons and holes from the bulk). In other words, the SPV is primarily caused by a variation in the charge density at the semiconductor surface/interface and that the total density of the acceptor and donor states is located at the interface. Accordingly, a general equation that describes two situations, steady-state condition and SPV decay, is obtained:

$$-\frac{N_s(0)}{2V_B\sqrt{1-\frac{SPV}{V_B}}}\frac{dSPV}{dt} = R_{bs}e^{\frac{SPV}{\eta K_B T}} - R_{bs} - R_{bs}^{*}$$ (eq. S4)

Where V_B is the band bending, $N_s(0)$ is the initial surface charge of the semiconductor, R_{bs} and R_{bs}^{*} are the bulk to surface flow of carriers in dark conditions and due to photo-generated electrons/holes, η is the ideality factor, K_B is the Boltzmann constant and T is the temperature.

Under continuous illumination the steady-state situation is achieved ($\frac{dSPV}{dt}$) and the SPV reach a “saturation” value (SPV_0) and the eq. S4 can be written as:

$$SPV_0 = \eta K_B T\, ln\left(\frac{R_{bs}^{*}}{R_{bs}} + 1\right)$$ (eq. S5)

Switching off the illumination, the SPV from the saturation value SPV=SPV_0 (at t=t_1=0), after a certain time (t_2), decreases to SPV=0. The bulk to surface flow of photo-generated electrons/holes stops, therefore R^{*}_{bs}=0. And from the eq. S4 it is obtained:

$$t = \frac{N_s(0)}{2V_B R_{bs}}\left(SPV(t) - SPV_0 + \eta K_B T \ln\left(\frac{e^{\frac{SPV_0}{\eta K_B T}} - 1}{e^{\frac{SPV(t)}{\eta K_B T}} - 1}\right)\right)$$ (eq. S6)

With:

$$R_{bs} = S_v N_v e^{-\left(\frac{SPV_0 + \Delta E_F}{K_B T}\right)}$$

Where S_v is the surface velocity recombination, N_v is the effective density of the valence/ conduction band, ΔE_F is the energy distance of the valence/conduction band (p/n doping) from the Fermi level.

Rearranging eq. S6 for the SPV effect to be dependent on time, we obtain:

$$SPV(t) = -\eta K_B T \ln\left(-\frac{1}{e^{\left[2V_B R_{bs}\left(\frac{t+\eta K_B T\, SPV_0}{N_S \eta K_B T}\right) - \ln\left(e^{\frac{SPV_0}{\eta K_B T}} - 1\right)\right]}} + 1\right)$$ (eq. S7)

Table S3 summarizes the best-fit parameters obtained by modelling of the SPV relaxation of the clean substrate and after CuPc deposition.

Sample	SPV (meV)	$N_s(0)$ (cm^{-2})	η	S_v ($cm\ s^{-1}$)
SiO_2/Si(100)	-95	4.23 x 10^{10}	0.63	4870
CuPc/SiO_2/Si(100)	-130	2.25 x 10^{10}	0.8	4450

Table S3. *Best fit parameters obtained by thermionic modeling of the SPV relaxation curves before and after CuPc deposition. SPV is the saturation value after laser excitation (t=0), $N_s(0)$ is the initial density of holes at the Si surface, η is the ideality factor and S_v is the surface recombination velocity.*

The initial surface charge (dark condition) and the transient surface charge (under illumination) in the semiconductor are related by:

$$N_s(SPV) = \sqrt{\frac{2(V_B - SPV)\varepsilon\varepsilon_0 N}{q}} = N_s(0)\sqrt{1 - \frac{SPV}{V_B}} \qquad \text{(eq. S8)}$$

with:

$$N_s(0) = \sqrt{\frac{2V_B\varepsilon\varepsilon_0 N}{q}}$$

Where ε is the static dielectric constant, ε_0 is the vacuum permittivity and q is the electron charge.

In the case of CuPc films, the inherent ultrafast nature of the singlet exciton CT[10,11] (< 1 ps) as compared to the rising of the SPV (hundreds of ps) allow us to approximate the CuPc film contribution to an additional source of charge density in the total balance of surface charges in the substrate (variation of $N_s(0)$). Accordingly we evaluated the number of additional surface charges required to observe an increase of the SPV signal from -95 meV to -130 meV (before and after CuPc deposition). The difference between N_s values before and after CuPc deposition represents the charge density transferred via CT, obtaining a theoretical value of 1×10^{10} cm^{-2}.

References:

(1) Ruocco, A.; Evangelista, F.; Gotter, R.; Attili, A.; Stefani, G. Evidence of Charge Transfer at the Cu-Phthalocyanine/Al(100) Interface. *Journal of Physical Chemistry C* **2008**, *112* (6), 2016–2025.

(2) Papageorgiou, N.; Ferro, Y.; Salomon, E.; Allouche, A.; Layet, J. M.; Giovanelli, L.; Le Lay, G. Geometry and Electronic Structure of Lead Phthalocyanine: Quantum Calculations via Density-Functional Theory and Photoemission Measurements. *Phys Rev B Condens Matter Mater Phys* **2003**, *68* (23).

(3) Evangelista, F.; Carravetta, V.; Stefani, G.; Jansik, B.; Alagia, M.; Stranges, S.; Ruocco, A. Electronic Structure of Copper Phthalocyanine: An Experimental and Theoretical Study of Occupied and Unoccupied Levels. *Journal of Chemical Physics* **2007**, *126* (12).

(4) J.F. Watts, J. Wolstenholme, Surface Analysis by XPS and AES, Wiley & Sons, Chichester, 2003.

(5) F. J. Himpsel, F. R. McFeely, A. Taleb-Ibrahimi, G. Hollinger, and J. A. Yarmoff. Microscopic structure of the SiO2/Si interface. *Phys Rev B* **1988**, 38 (9).

(6) F. J. Himpsel, B. S. Meyerson, F. R. McFeely, J. F. Morar, A. Taleb-Ibrahimi and J. A. Yarmoff, in *Proceedings of Enrico Fermi School on "Photoemission and Adsorption Spectroscopy of Solids and Interfaces with Synchrotron Radiation"*, eds. M. Campana and R. Rosei, Amsterdam, 1992.

(7) S. M. Sze, *Physics of Semiconductor Devices (Sze - 2nd edition),* New York, 1981.

(8) M. Krzywiecki PhD Thesis "Studies of CuPc ultra-thin layers deposited on Si(111) native substrates" Institute of Physics–CSE, Silesian University of Technology, Gliwice, Poland, Gliwice 2010 and refs therein.

(9) Reshchikov, M. A.; Foussekis, M.; Baski, A. A. Surface Photovoltage in Undoped N-Type GaN. *J Appl Phys* **2010**, *107* (11).

(10) Baek, D.; Rouvimov, S.; Kim, B.; Jo, T. C.; Schroder, D. K. Surface Recombination Velocity of Silicon Wafers by Photoluminescence. *Appl Phys Lett* **2005**, *86* (11), 1–3.

(11) Dutton, G. J.; Robey, S. W. Exciton Dynamics at CuPc/C 60 Interfaces: Energy Dependence of Exciton Dissociation. *Journal of Physical Chemistry C* **2012**, *116* (36), 19173–19181.
